# Peer support provider and recipients’ perspectives on compassion in virtual peer support stroke programs: “You can’t really be supportive without compassion”

**DOI:** 10.1371/journal.pone.0309148

**Published:** 2024-10-04

**Authors:** Hardeep Singh, Michelle L. A. Nelson, Meera Premnazeer, Arta Taghavi Haghayegh, Sarah Munce, Christina Sperling, Carolyn Steele Gray

**Affiliations:** 1 Department of Occupational Science & Occupational Therapy, Faculty of Medicine, University of Toronto, Toronto, Ontario, Canada; 2 The KITE Research Institute, Toronto Rehabilitation Institute-University Health Network, Toronto, Ontario, Canada; 3 Rehabilitation Sciences Institute, Temerty Faculty of Medicine, University of Toronto, Toronto, Ontario, Canada; 4 Science of Care Institute, Lunenfeld-Tanenbaum Research Institute, Sinai Health System, Toronto, Ontario, Canada; 5 Institute for Health Policy, Management and Evaluation, University of Toronto, Toronto, Ontario, Canada; 6 March of Dimes Canada, Toronto, Ontario, Canada; Almoosa College of Health Sciences, SAUDI ARABIA

## Abstract

**Background:**

Peer support programs demonstrate numerous benefits, including emotional, instrumental, informational, and affirmational social support. Since the COVID-19 pandemic, many peer support stroke programs in Canada have been delivered virtually. Compassion must be consistently applied to build meaningful interactions, but the shift to virtual services may have changed the quality of interaction and compassion in virtual services. While compassion is recommended in health and social services to improve outcomes, satisfaction, and service quality, compassion in virtual peer support stroke programs remains understudied. We aimed to describe compassionate support in virtual peer support stroke programs from peer support providers’ and recipients’ perspectives.

**Methods:**

This qualitative descriptive study was guided by Sinclair & colleagues’ model of compassion. Peer support recipients or peer support providers participated in interviews transcribed and analyzed using a hybrid thematic analysis.

**Results:**

Sixteen were peer support recipients, six were peer support providers, and two were both peer support providers and recipients. Participants agreed that compassion was essential in these programs. Participants perceived compassion to be a result of the virtues of compassionate facilitators (i.e., genuineness, passion, and empathy), relational space, and communication within the virtual peer support stroke program (e.g., sense of awareness or intuition of compassion, aspects of engaged peer support provision), virtuous response (e.g., knowing the person and actions that made the peer support recipient feel like a priority). Compassion was facilitated by listening and understanding peer support recipients’ needs as they relate to stroke (i.e., seeking to understand peer support recipients and their needs), attending to peer support recipients’ needs (e.g., timely actions to address their needs), and achieving compassion-related program outcomes (e.g., alleviating challenges and enhancing wellbeing). The absence of these components (e.g., lacking genuineness, passion and empathy) was a barrier to compassion in virtual peer support stroke programs.

**Conclusions:**

Study findings describe facilitators and barriers to perceived compassion in virtual peer support stroke programs and provide practical recommendations that can be adapted into programs to improve program quality.

## Introduction

Many community-based health and social programs, including peer support stroke programs, have been offered virtually during the COVID-19 pandemic [1, 2]. Peer support is provided by and to individuals with shared experiences [3]. Peer support stroke programs demonstrate benefits, including decreased loneliness and sense of isolation, enhanced community reintegration, and acceptance of chronic conditions [3, 4–6]. The Canadian Stroke Best Practices recommend peer support stroke programs to support the psychological, emotional, and social needs of individuals living with chronic stroke, as they provide emotional, instrumental, informational, and affirmational social support to others with a shared experience [3, 7].

Virtual peer support groups are offered in various formats [8], including asynchronous (e.g., forums and discussion boards) [9] and synchronous (e.g., online meetings and workshops) [10]. Synchronous programs where participants attend at the same time provide an opportunity to capture visual/non-verbal communication (e.g., facial expressions). The benefits of virtual peer support are well-recognized [11, 12], and include low cost, overcoming geographical distance/boundaries and transportation barriers, reaching individuals at a broader scale, and accommodating individuals with limited mobility [11, 12]. Reach is particularly relevant in the context of stroke because over one million individuals will be living with stroke across Canada by 2030 [13]. In addition, chronic stroke symptoms have increased over the past decade, resulting in more individuals living with long-term challenges after a stroke, such as depression, anxiety, decreased community participation, social isolation, and communication difficulties [14–16]. Thus, there is an increasing demand for high-quality community-based stroke services, like peer support, that can reach broad audiences [17].

While virtual peer support programs appear well-suited, there are concerns regarding the decreased effectiveness compared to similar in-person interventions [18, 19]. For instance, there may be a decreased quality of interpersonal relations within these programs, as studies have shown that individuals may have weaker connections and relationships with other group members compared to in-person programs [20]. Moreover, these programs do not allow for haptic connections (e.g., handshakes, hugs), which have positive effects in health contexts [20]. Touch is also often an important part of therapeutic care for people who have experienced a stroke [21]. Studies speak to virtual support as playing a complementary role rather than a substitute for hands-on interactions [21, 22]. Despite the limited physical interactions, the virtual environment can help foster social support, social networking, and aid in providing recovery education [23].

Compassion is defined as a “concern for the suffering or unmet need of another, coupled with the desire to alleviate that suffering” [21]. Compassionate care is delivered by creating a safe and comfortable environment for interaction that allows providers to have a good understanding of the service users and their needs [22]. Compassion is widely encouraged in health and social services as it improves client outcomes, satisfaction and service quality [23–26], and is considered a hallmark of high-quality care in health services [27], which includes rehabilitation and stroke care [28]. Individuals with stroke have indicated the need for compassionate peer support providers [22, 29]. Peer support providers engage in peer support programs to offer *compassion* and empathy towards others, while peer support recipients engage in these programs to obtain support [6, 30, 31]. A scoping review of web-based peer support interventions found that increased compassion results in positive outcomes from the interventions [32]. As such, compassion towards others and connectedness with peers are the goals of peer support programs [33].

A shift to virtual delivery of peer support programs may impact compassion within peer support, however, compassion within virtual peer support programs is understudied. While some evidence suggests that virtual interactions can be adapted to deliver personalized support and compassion can be taught to facilitators [34], the compassion-based needs of individuals within a virtual context must first be understood [34]. A poor understanding of compassion within virtual services could lead to uncompassionate service delivery and poor relationships [22]. Despite compassion being a critical aspect of peer support, as it enables a sense of connectedness and understanding about shared lived experiences and is a facilitator of compassion [22], it is currently understudied in virtual programs [35–42], including peer support stroke programs.

To address current research gaps and generate a better understanding of compassion [22], the objective of this study was to describe compassionate support, including facilitators and barriers of compassion, in virtual peer support stroke programs from the perspectives of stroke peer support providers and recipients.

## Materials and methods

### Design

We conducted a qualitative descriptive study [43]. A qualitative descriptive approach was appropriate to study compassion in virtual peer support stroke programs because little is known about this topic. This qualitative approach aligned with our aims, allowing us to interpret and describe participants’ subjective perspectives and experiences of compassion in virtual peer support stroke programs [44]. REB approval was obtained from the University of Toronto Research Ethics Board (#43,096). All methods were carried out in accordance with relevant guidelines and regulations, including verbal informed consent obtained from all participants and the consent process was documented by the researcher obtaining consent.

### Research framework: Elements of compassion

In 2016, Sinclair and colleagues [27] described the following elements of compassion based on the perspectives of individuals receiving palliative care services; the definitions of these elements were adapted by the lead author to fit our study population (i.e., peer support providers and recipients) and context (i.e. virtual peer support stroke programs) (see Table 1). This model guided our data collection and analysis as it enabled us to explore whether/which elements of compassion applied to the current study’s participants and context.

**Table 1:** Sinclair & colleagues’ key elements of compassion adapted to fit the current study [27].

Sinclair & colleagues’ categories of compassion and brief descriptions	Adapted themes relating to each category
1: Virtues *Virtues of peer support facilitators that predicted compassion*	Understanding
	Love
	Tolerance
	Acceptance
	Kindness
	Genuineness
	Openness
	Honest
	Authenticity
	Care
2: Relational Space *Relational space refers to the context/content of the virtual peer support stroke program*	Peer support recipients’ awareness or intuition/sense of providers’ compassion
	Engaged peer support provision: any tangible indicators of compassion from peer support providers during the virtual peer support stroke program
3: Virtuous Response *What was done to demonstrate the virtues*	Knowing the person: peer support recipients approached as individuals and their challenges/concerns s
	Person as priority: prioritizing the person’s needs before own assumptions or program priorities
	Beneficence: wanting the best for the peer support recipient
4: Seeking to understand *Understanding individuals and their needs*	Seeking to understand the person: doing things to get a deeper understanding of the peer support recipient and their experience
5: Relational communicating *Verbal or nonverbal demonstration of compassion*	Demeanor: compassion-based non-verbal communication
	Affect: resonating with peer support recipient’s emotions
	Behaviours: interpersonal skills of peer support providers that depicted compassion, such as listening and showing respect
	Engagement: how much peer support recipients felt peer support providers were involved in the program and recipients’ involvement in the program
6: Attending to needs *Attending to and addressing compassion-related needs*	Compassion related needs: what needs participants felt compassion alleviated/decreased
	Timely: addressing suffering in a timely and responsive manner
	Action: tangible process to reduce/alleviate suffering
7: Reported outcomes *Impact of compassion*	Alleviates suffering: compassion alleviated suffering
	Enhances wellbeing: compassion enhanced wellbeing
	Enhances care: compassion enhanced an aspect of care

### Participants

Purposeful sampling [45] was used to selectively recruit people across Canada who have had a stroke and were either: i) a peer support recipient (i.e., adults with stroke who received virtual peer support) or ii) a peer support provider (i.e., adults with stroke who provided virtual peer support). Given their key role in virtual peer support stroke programs, both peer support recipients and providers were included in this study. In addition, we sought to include both perspectives to potentially uncover nuances in experiences and perceptions of compassion in the programs. Participants were recruited through electronic advertisements shared on social media and within peer support stroke programs by the research team and community organizations, including organizations that deliver peer support stroke programs (e.g., March of Dimes Canada, Evergreen Therapy). Recruitment and data collection continued concurrently until data sufficiency was attained [45] between August 1, 2022, to February 16, 2023. Data sufficiency was determined to be the point at which HS, ATH and MP agreed that the data were rich enough to address the study objective [45].

### Data collection

A semistructured interview guide based on the elements of compassion [27] and informed by prior literature [46, 47] was used to explore elements of compassion in a virtual environment, including enablers and barriers to compassion (see S1 Appendix). Semi-structured interviews helped us to explore participants’ viewpoints while ensuring interviews focused on the research aim [48]. Consistent with qualitative descriptive methodology, the interview guide was iteratively modified during data collection to probe new ideas related to compassion within virtual programs [27]. Interviews were conducted by HS, MP, and ATH on Zoom or by phone [49]. The interviews were individually adapted to support participants in adapting communication using strategies suggested for communicating with individuals with aphasia [50–52], as our research team included individuals trained in facilitating conversations with individuals with aphasia. For example, some participants were provided with interview questions before their interview, and Zoom chat was used for part or all interviews for those who preferred written communication.

### Data analysis

Interviews were audio-recorded on Zoom and de-identified files were stored on a secure, ethics-approved server. The audio-recordings were transcribed in Microsoft Word, with transcripts checked for accuracy by MP and ATH. A hybrid thematic analysis approach was used to analyze interview data [53]. First, HS and MP independently conducted data-driven coding for three transcripts using NVivo software. Using constant comparative techniques, they compared the codes from the first transcript to the subsequent transcripts. Team discussions were used to compare codes between coders and generate an amalgamated codebook. HS grouped the codes that addressed the research objective into higher-level categories and coded the remaining transcripts using the amalgamated codebook. HS categorized the inductive codes according to the adapted elements of compassion framework [27]. Inductive codes that did not align with the elements of compassion were added as new components to the framework. Data from peer support providers and recipients were compared to identify whether there were any differences in their perspectives. In addition, we organized the facilitators and barriers to compassion according to the following categories: peer support facilitator-related, peer support recipient-related and program-related. Lastly, the themes were reviewed by MP and ATH to ensure they were representative of the data (i.e., triangulation). The qualitative reporting standards called ‘Standards for Reporting Qualitative Research’ were used to improve reporting transparency [54].

## Results

Of the 24 participants included in this study, 16 were peer support recipients, 6 were peer support providers, and 2 were both a peer support provider and the recipient (Table 2-participant characteristics). Interviews averaged 61 minutes (SD: 22), with the shortest interview being 12 minutes, but most (58%) lasted over an hour. Longer interviews allowed deeper exploration of study topics, an opportunity for breaks to minimize fatigue symptoms and the interview to proceed at a slower pace if required by a participant to accommodate their communication preferences. Interviews were conducted between August 2022 to February 2023.

**Table 2:** Participant characteristics (n = 24).

	Peer support recipients (PSR; n = 16)	Peer support providers (PSP; n = 6)	Both (peer support provider and recipient; n = 2)
*Average age*, *range (years)*	60.8, 25–88	65.3, 50–76	58.5, 42–75
*Gender (e*.*g*., *Man; Woman; Non-binary/non-conforming; Transgender; Other)*	Woman: 10 Man: 6	Woman: 3 Man: 3	Woman: 1 Man: 1
*Place of birth*	Canada: 13 Other: 3 (Germany, India, Pakistan)	Canada: 5 Other: 1 (UK)	Canada: 2
*Self-reported ethnicity*	Mixed heritage (German, Irish, Indigenous): 1 White-North American: 10 South Asian: 2 Polish: 1 First Nations: 1 White-European: 1	White-North American: 2 British-Irish: 2 British Canadian: 1 Jewish: 1	White-European: 1 White-North American: 1
*Location*	Suburban: 5 Rural: 3 Urban: 8	Suburban: 4 Rural: 1 Urban: 1	Rural: 2
*Highest level of education*	Less than high school: 2 Some college or university undergraduate: 2 College or University undergraduate: 11 Graduate degree: 1	College or university undergraduate: 4 Graduate degree: 1 Post-graduate: 1	Less than high school: 1 College or university undergraduate: 1

### Participants’ description of virtual peer support stroke programs

Participants indicated that virtual peer support stroke programs were generally conducted on the Zoom platform, with a few on the Ontario Telemedicine Network platform. Participants explained that the programs were either group-based, with approximately 25–70 other members, or one-on-one. The group programs were facilitated by peer support providers who had a stroke along with program staff (i.e., staff members and volunteers without stroke), and a peer support provider with stroke facilitated the individual programs. Participants explained that group-based programs used the Zoom breakout function to sort the participants into smaller groups of approximately 10 to 15 people, which helped ensure that all members had time to participate in the discussion.

Participants indicated that virtual peer support stroke programs were generally ongoing and covered various topics, including difficult subjects related to living with stroke, medical issues, how to prevent another stroke, “living with the physical changes,” driving, and food preparation. Some programs involved leisure or social activities, such as bingo, and others were therapeutic (e.g., exercise) or educational (e.g., discussions and presentations).

### Compassion in virtual peer support stroke programs

All participants indicated that compassion was a required component of virtual peer support stroke programs. According to the participants, compassion was a result of the virtues of compassionate facilitators (theme 1), relational space and communication within the virtual peer support stroke program (theme 2), virtuous response (theme 3), compassion means listening and understanding peer support recipients needs as they relate to stroke (theme 4), attending to peer support recipients’ needs (theme 5), and compassion-related program outcomes (theme 6). Table 3 provides a summary of the themes and subthemes of our hybrid thematic analysis. In addition, Table 4 presents a summary of the facilitators and barriers to compassion identified from the study findings.

**Table 3:** Summary of the themes and subthemes derived from our hybrid thematic analysis aligned to the adapted Sinclair & colleagues’ elements of compassion.

Themes	Subthemes derived from the hybrid thematic analysis *inductively coded
**Theme 1: Virtues of compassionate facilitators** *Virtues of peer support facilitators that predicted compassion*	*Genuineness*
	Passion*
	Empathy*
**Theme 2: Relational space and communication within a virtual peer support stroke program** *Relational space and communication were the context/content of the virtual peer support stroke program*, *and compassion displayed through verbal and non-verbal communication*	*Sense of awareness or intuition of compassion*
	*Aspects of engaged peer support provision*
	Compassionate verbal and non-verbal communication*
**Theme 3: Virtuous response** *Virtuous response referred to what was done to demonstrate the virtues*	*Knowing the person*
	Actions that made the peer support recipient feel like a priority*
	*Beneficence*
Theme 4: Compassion means listening and understanding peer support recipients needs as they relate to stroke* Seeking to understand referred to understanding individual and their needs	Seeking to understand peer support recipients and their needs*
**Theme 5: Attending to peer support recipients’ needs** *Attending to needs referred to compassion-related needs and how they were addressed*	*Compassion-related needs*
	*Timely*
	*Action to address their needs*
**Theme 6: Compassion-related program outcomes** *Outcomes referred to the impact of compassion*	Alleviating challenges*
	*Enhancing wellbeing*
	Enhancing support*

**Table 4:** Facilitators and barriers to compassion identified by peer support recipients and providers in virtual peer support stroke programs. Facilitators of compassion in virtual peer support stroke programs perceived by peer support recipients and providers. Barriers to compassion in virtual peer support stroke programs perceived by peer support recipients and providers.

Table 4. Facilitators and barriers to compassion identified by peer support recipients and providers in virtual peer support stroke programs Facilitators of compassion in virtual peer support stroke programs perceived by peer support recipients and providers	Barriers to compassion in virtual peer support stroke programs perceived by peer support recipients and providers
**Peer support facilitator-related** • Peer support facilitators demonstrating genuineness, passion, and empathy • Peer support facilitators with lived experience of stroke or have compassion towards others and knowledge of stroke • Peer support facilitators actively listening to peer support recipients, encouraging all members to engage in the discussions/activities • Peer support providers and recipients appearing engaged in discussion by learning/sitting forward • Peer support providers verbally describing their challenges to establish a connection with other participants • Balancing sharing enough details but not too much • Effective re-direction: Not rushing or interrupting peer support recipients while they are sharing and reassuring individuals who experienced cognitive or communication challenges • Sharing resources and advice to alleviate stroke-related challenges • Timely follow-up from peer support facilitators • Peer support facilitators modifying programs based on participant needs/preferences	**Peer support facilitator-related** • Peer support facilitators lacking genuineness, passion, and empathy • Peer support facilitators defensive to feedback and not able to relate to individuals with stroke • Inattentive peer support facilitators who failed to recognize participants who did not have an opportunity to speak during the session • Peer support providers share too many details about themselves • Poor facilitation and re-direction: Rushing participants • Peer support facilitators reading off of a script • Peer support facilitators/providers appearing disengaged in virtual discussions by sitting back in the chair
**Program structure-related** • Use of the speaker spotlight function on Zoom to allow participants to view the speaker’s facial expressions and demeanour • Use of Zoom’s raise hand function which reduced interruptions • Structured agenda/discussion topics sent by email before the program (preferred by some)	**Program structure-related** • Technical support to help with the use of the virtual platform • Program matching participant goals, e.g., unstructured programs (not preferred by some) • Privacy rules restricting sharing of physical resources • Not being in the same physical space as others
**Peer support recipients-related** • Personalized interactions with other peer support recipients • Calm and happy demeanour of peer support recipients • Verbal expressions of actions or demeanour e.g., stating ‘virtual pat on the back’ or ‘virtual hug’ or thumbs up emojis to indicate agreement • Peer support recipients feel heard and understood	**Peer support recipients-related** Noises or conversations in the background within other participants’ environments Lack of physical contact with other members

### Theme 1: Virtues of compassionate facilitators

Peer support recipients in a virtual peer support stroke program provided examples of the three virtues that they perceived exemplified compassion: genuineness, passion, and empathy. According to peer support recipients, PSR13 and S4, facilitators who genuinely cared for the wellbeing and feelings of peer support recipients were compassionate. S4, a peer support recipient, described genuineness as the ability to recognize the impact of one’s words on others and address communication errors, “if you make a mistake or you say something the wrong way, or you see someone’s expression or something. You have to have that self-confidence to say, did I just say something the wrong way or that bothered you? So I think it’s just being genuine.” Peer support recipients perceived that being genuine was an innate trait that “you either are or aren’t” (S4) rather than something that could be taught. S6, a peer support recipient, expressed that compassionate facilitators conveyed a passion for the program and facilitation and appeared to truly want to be part of the program, which recipients could easily sense. Additionally, peer support recipients, such as PSR13, felt that *compassionate* facilitators and other peer support recipients were empathetic in that they recognized the unique stroke experiences of peer support recipients, while non-compassionate facilitators were someone that was not nice or were just “doing it for the money.” Moreover, when facilitators were defensive to feedback, it signified to S6, a peer support recipient, that the facilitator was not compassionate: “the person is being a little bit defensive, the one who’s facilitating. So, I think that like signifies that she’s not being compassionate enough”.

### Theme 2: Relational space and communication within a virtual peer support stroke program

This theme captured participants’ descriptions of the content and context of compassionate encounters with facilitators and other program members. Peer support recipients discussed three subthemes: their sense of awareness or intuition of compassion (subtheme 2a), aspects of engaged peer support provision (subtheme 2b), which were sensed by facilitators’, and compassionate verbal and non-verbal communication, such as others’ demeanour, affect, behaviours and engagement (subtheme 2c).

#### Subtheme 2a sense of awareness or intuition of compassion

Peer support recipients described that they could sense compassion in others, even in a virtual setting. This sense was developed in response to interactions with others in the virtual setting. Communication with others allowed PSR1, a peer support recipient, to differentiate between genuine and ingenuine gestures: “You can actually like *feel* like maybe this is like wanting to be like, a genuine, like feeling between this person and you”.

Peer support providers agreed that compassion was something that others could feel in response to their ability to communicate effectively and emotionally connect with others. To create meaningful connections, S3, a peer support recipient, recommended that facilitators should be someone with a stroke who can meaningfully relate to peer support recipients: “it would be better to have a coordinator with stroke…they can understand what the- what the life is about.” S4, a peer support provider, explained that she built an emotional connection with other recipients by putting herself in their shoes, “I think for me it’s…all always about educating yourself about what happens with a stroke and if you can find any little bit of it that you can try to relive yourself. Try to put yourself in their shoes.” Similarly, S5, who had a lived experience of stroke, emphasized the value of her ability to relate with peer support recipients living with stroke on a personal level:

I can read their minds in a way–and that’s another thing–I say things out loud, that they’re thinking that they’re too afraid to say out loud or that they don’t want to say out loud. Or sometimes, it’s not even- They can’t even put those words, they can’t even form the words, but I say them and they’re like: ‘Yeah! That’s how I feel!’

#### Subtheme 2b aspects of engaged peer support provision

Participants provided examples of engaged peer support provision that fostered a compassionate environment through the actions and behaviours of others. Peer support providers indicated that they created a compassionate virtual space through their genuine desire to support others, actively listening to peer support recipients, and ensuring that all peer support recipients had an opportunity to participate in the virtual group discussion. To engage each peer support recipient in the discussions, providers tracked who had or had not participated in the discussion and encouraged those who had not spoken and quieter individuals to speak using their names.

For peer support recipients, meaningful engagement meant the ability to discuss their challenges (or suffering) with other members of the program; however, not all agreed they were suffering. Four participants described the challenges of this meaningful engagement in a virtual environment due to their lack of comfort with Zoom and inattentive facilitators who failed to recognize participants who did not have an opportunity to speak during the session. According to S8, a peer support recipient, this created an “uncompassionate environment”, preventing some recipients from sharing their needs. In addition, limited interactions and connections with other members individually created a challenge to engage in virtual peer support programs and missed opportunities for compassionate care.

Peer support providers reflected on the strategies they used to create compassion through engagement with peer support recipients in virtual environments, which were also crucial for relationship building in a virtual environment. For instance, some providers, like PSR4, a peer support provider and recipient, explained that they did not stick to a script and tried to personalize interactions with other peer support recipients: “I’m not textbook. Like reading out of a textbook, like…I’m really involved with them.” Additionally, S8, a peer support recipient recommended that peer support providers could engage others by introducing themselves and sharing their stroke experience, including the challenges they experienced:

“The first thing I would do is introduce myself, but then…I would give them a brief synopsis, a brief overview of what happened to me when I had my stroke. What type of stroke and then what deficits that it left me with physically and mentally…[I would say] I’m going to listen to you, but I just wanted to give you my background.”

S8 indicated that this introduction was important so that peer support recipients could relate to the providers’ feelings and experiences. In sum, for a compassionate environment to be created, it was important for facilitators and peer support recipients to be engaged in the sessions.

#### Subtheme 2c compassionate verbal and non-verbal communication

Peer support recipients explained that demeanor (e.g., tone of voice, eye contact) of others, including peer support providers, facilitators and other peer support recipients, contributed to the perception of compassion. They described a compassionate demeanor as “calm and not yelling” and someone happy to see them. Peer support recipients agreed that maintaining eye contact (e.g., looking directly at the camera) and nodding to indicate agreement were demeanors suggestive of compassion in a virtual environment. In terms of interactions with other peer support recipients in the program, peer support recipients explained that the speaker spotlight function on virtual platforms enabled a compassionate environment as it allowed them to have a better visual of the speaker’s facial expressions and demeanor. PSR1, a peer support recipient, explained that the spotlight function also hid the view of other recipients, reducing their concerns about others reacting negatively to what they shared; as a result, they were able to open up more than they would in an in-person setting:

I think it’s just you were able to open up more when it’s not in a program setting, like with people around you, because you’re basically just focusing on the one piece in the middle…I think you can pick up more people’s body um, what’s that called? Body languages because when you’re in a program setting on Zoom or whatever it’s you just see their faces, but the only one that you’re really focusing on is the person that’s doing it, right? So you don’t see everybody, like if they’re making faces or something.

PSR3, a peer support recipient, explained that Zoom’s raising hand function helped recipients feel respected and fostered compassion as they felt they were not interrupted when sharing their needs. In addition, peer support recipients highlighted the importance of engaging peer support providers and program staff, as they could tell which facilitators were engaged and attentive to the group discussions and dynamics. At the same time, participants could easily identify when someone was or was not paying attention to them, even in a virtual context. As a peer support provider, PRP1 was conscious about his body language and cautioned that in a virtual environment, sitting too far back suggested an uncompassionate demeanor, whereas if you sit forward, you appear more engaged and compassionate: “I mean, there’s a lot of people just sit way back in a chair and, you know, they’re not participating.” Finally, participants indicated using emojis to demonstrate gestures, such as thumbs-up emojis, to convey their demeanor.

### Theme 3: Virtuous response

This theme captures participants’ descriptions of how facilitators and other peer support recipients demonstrated the virtues of compassion described in theme 1. Virtuous responses were demonstrated by getting to know the person (subtheme 3a), through actions that made peer support recipients feel like a priority (subtheme 3b), and through beneficence (subtheme 3c).

#### Subtheme 3a knowing the person

Peer support recipients felt a sense of connectedness and compassion when they could talk to others about topics beyond their stroke experiences. For instance, when others took an interest in their personal life events or geographical location (e.g., province, city), peer support recipients in group-based virtual peer support stroke programs resided across Canada. PSR9, a peer support recipient, shared an example where he felt like part of a family due to strong connections with others. This connection was demonstrated when everyone in the program took an interest in another participant’s newborn baby.

However, some peer support recipients expressed concerns about the safety and security of a virtual environment, which decreased perceived compassion due to feeling less comfortable and open to sharing their stories and challenges. For instance, participants described feeling uncomfortable when they heard people speaking in the background of another group member’s environment because they did not know who else was listening to them. As a result, peer support recipients such as PSR13, were less likely to share their concerns and challenges during the program, which hindered sharing their experiences.

I didn’t really wanna air dirty laundry in front of other people. And that’s just a me thing. Did I feel? Safe enough to do it, I could have. But I didn’t. I guess I didn’t feel 100% so. It made it hard. When you are online and you don’t also know who else is in the room.

Peer support recipients felt that in a virtual environment, the lack of communication cues from others also made it hard for peer support them to establish a rapport or feel comfortable sharing their thoughts and feelings. PSR1 suggested that it may be easier opening up to others in an in-person program.

I think [building rapport] might have been like a little longer, because you don’t know -when in person you can judge whether or not you like a person or not. With their online all you see is like their voice, right? And then how their eyes is compassion.

They noted that the peer support provider’s ability to relate to them was essential for building trust and rapport and demonstrating compassion. However, the lack of visual cues in a virtual environment made it challenging because they could only see a limited amount of a person’s body (e.g., chest-up), so they did not see the full spectrum of visual cues. S5, a peer support provider, shared that verbally describing their physical challenges was necessary to establish a connection with the group because others could not see it related to him:

They could see the way I walked. So, if they had physical deficits, they could compare themselves to me in their own head and, you know?…another woman said to me ‘I didn’t know- I read about hope in the books, but I didn’t know what hope looked like until you walked in the room.’ Now, can I do that virtually? I, for the most part I can, the physical piece is missing where someone sees the way you walk, they would look at your gait, they see your arm, they see, you know, so that sort of flash–the picture is missing, but I can describe it as well.

However, there was a fine balance between sharing enough details and sharing too much, as some peer support recipients, such as S4, felt that facilitators who shared too much were less compassionate because they focused the session on their own experiences and challenges rather than the needs of peer support recipients:

While the co-facilitator is a survivor, she’s not obviously very compassionate because she’s been there. What I struggled with her was I didn’t think she had a good grasp of when to share her personal stories and when to let the program share theirs? OK, there was times when I felt like why are we focusing so much on you?..People are compassionate, there’s no two ways about it, but in the bigger program the community program I think you could really see the lack of facilitation skills in people not being able to stay on topic. People, people bringing their own agenda.

#### Subtheme 3b actions that make peer support recipients feel like a priority

Participants described feeling valued and prioritized when they had an opportunity to meaningfully participate in discussions without being rushed or interrupted. Both peer support recipients and facilitators agreed that facilitators had a critical role in ensuring that everyone had an opportunity to participate in the program. However, in a virtual context, some participants noted it was challenging for facilitators to redirect peer support recipients, especially those with aphasia, without interrupting or rushing them. As a peer support recipient with aphasia, PSR12 shared the importance of allowing recipients to speak freely: “As far as compassion is allowing the survivor to talk, hear, let them get it off their chest as much as they can, and if we go off a tangent, which I know I’ve done a few times…Avoid pointing out our weaknesses.” As a peer support provider, S5 shared her approach to making participants feel respected and prioritized (i.e., comfortable with sharing their stories) by recognizing and reassuring them if they were experiencing communication difficulties:

Cognitive difficulties were, and so, when somebody would- They’d start, and then they would lose their train of thought- So they would start something, and then they’d go ‘Ahh-’ and then they’d lose their train of thought, I’m like ‘Take your time.’ I’d sort of offer the same advice, ‘Don’t worry, take your time, we’re not going anywhere.’

Another peer support provider, S7, shared her introductory speech to the group with which aimed to make peer support recipients feel prioritized from the beginning of the program: “Well, I think I’d go online first of all, and just talk to them person to person and say ‘Welcome. I want you to know we’re- Everybody here is in the same boat and we’d like you to feel free to join when your comfortable, and if there’s anything I can do to help you, let me know.’” Moreover, a peer support recipient appreciated receiving a structured agenda or discussion topic by email before the program, as suggested to them that the program prioritized them and respected their time.

Peer support recipients also described aspects of the virtual peer support stroke program that made them feel less prioritized. While some enjoyed the group-based format, the large program capacity of the virtual sessions made PSR3 feel less important or prioritized: “it makes you feel less, you know, I guess important in a way.” Similarly, peer support providers agreed that virtual programs with larger capacities made it difficult to facilitate the program well, impacting how heard/prioritized other members felt during the program. PRP1, a peer support provider, spoke about the challenges of facilitating a large virtual program due to her aphasia and technical challenges using Zoom:

When you’re on your virtual ones and all of a sudden there’s, you know, 40… 40 pictures all around you, and it’s hard to see who’s talking. Even if you say, put your hand up, you know, you put that yellow thing up to talk, people don’t. They just start talking because people that communicate, we talk how we feel immediately… As a chair, I had to always control it, and that was difficult for some people, especially for me. It was difficult ‘cause I- I have an aphasia problem anyway.

Finally, peer support recipients felt less important or prioritized when others interrupted them (e.g., did not use the raise hand function on Zoom) or did not recognize that they may need more time to articulate their thoughts due to verbal or cognitive challenges.

#### Subtheme 3c beneficence

Participants emphasized the importance of beneficence in virtual peer support stroke programs. Peer support recipients described beneficence as a demonstration of a genuine desire from others for them to “get better.” Both peer support providers and recipients exemplified beneficence towards other peer support recipients by sharing personal advice and directing recipients to resources that may assist their recovery. However, a few peer support recipients expressed a desire to do more to help a peer support recipient but were limited by virtual interactions. Limitations in virtual interactions such as not being in the same physical space as others and privacy rules restricted the individual from sharing physical resources with other members. PRP2 explained, “I can’t really send stuff to them, you know, which is a disadvantage. To some degree, you know, just because of the way it’s set up, I don’t have the email address…telephone”.

### Theme 4: Compassion means listening and understanding peer support recipients needs

This cross-cutting theme had some overlap with earlier themes. There was a consensus among participants about the importance of compassion in virtual peer support stroke programs. Participants emphasized that compassion was demonstrated by facilitators and other recipients actively listening to them and allowing them to feel heard. This theme was enabled by facilitators’ virtues (theme 1), relational space within the virtual peer support stroke program (theme 2), and virtuous response (theme 3). Peer support providers highlighted the importance of recognizing peer support recipients as individuals and understanding their unique needs for compassionate encounters. S5 (peer support provider) explained that validation of the recipient’s feelings was just as critical as listening to the peer support recipients’ concerns. Both PSR13 and PSR3 (peer support recipients) explained that if a participant was “brave enough to speak out,” facilitators should “make sure [they] were heard completely” and their concerns were not dismissed. PRP1, a peer support provider, shared that he created a compassionate program by understanding the needs and feelings of others. He explained that he could feel “more for people with physical problems even though I don’t have a physical problem” and that they could “help each other.” Participants emphasized that it was not necessary to have the same experiences or challenges to emphasize with another person. For example, PSR1, a peer support recipient, stated that “they didn’t experience what you was going through, but they would still have the knowledge or background through someone else’s story or relationship that they were able to present it and just be a compassionate, caring person this way.” Finally, PRP2, a peer support provider, highlighted the importance of making peer support recipients feel heard and their needs understood from the program’s start as a means of demonstrating compassionate support:

The first thing that I would do in that conversation is tell me about your situation. What deficits do you have for them? So when did you have your stroke? So you’re really connecting with them on a more personal basis. Like you’re finding out both where they’re at and what they’re struggling with. Before you start trying to give them a place. We’ll find out really where they’re at and I think that that’s crucial in my estimate.

### Theme 5 attending to peer support recipients’ needs

This theme describes compassion in response to peer support recipients’ compassion-related needs (subtheme 5a), timely response to their needs (subtheme 5b), and actions to address their needs (subtheme 5c).

#### Subtheme 5a compassion-related needs

Compassion-related needs were described as individualized, with peer support recipients indicating that they were less likely to engage in virtual peer support stroke programs if the program failed to meet their expectations and needs. Participants were dissatisfied with programs that did not address their needs. For example, S7, a peer support provider, stated that she sought “very specific information” and did not feel like she received that information, leading to feelings of depression. Preferences for programs’ structure and format varied among participants, with some preferring structured discussion, while others preferred unstructured participant-led discussions, where “any topic was fair game”.

Additionally, participants also indicated they missed physical contact during virtual programs, which resulted in difficulties in attending to emotional needs in a virtual environment. PSR3, a peer support recipient, expressed a desire for physical contact with other peer support providers who “could help support me, like give me like a hug and stuff. So that would have been nice.” Similarly, PRP1, a peer support provider, described that it could be hard to attend to emotional needs in a virtual environment: “Where you got a tear, someone said ‘a tear is coming down here your cheeks’ you know? It’s very hard…virtual can be sometimes cold because we don’t feel that emotion.” However, virtual programs were still perceived to address some compassion-related needs, such as feeling understood, sharing resources to alleviate challenges, and reducing isolation.

#### Subtheme 5b timely

Participants described the importance of timely follow-up from facilitators or peer support recipients when they were experiencing difficult life challenges. PRP1, a peer support provider, exemplified the following up with a peer support recipient when they shared some difficult news during the program:

Someone said ‘my grandmother just passed away’ or someone is saying something very sad or upsetting…this is somebody just sort of goes off, the screen is off, which means that we might phone them the next day and say, you know, I know you were cut off. Was there a mechanical problem or is something really bothering you? And then we have an opportunity to talk to you.

PSR1, a peer support recipient, appreciated knowing they could receive timely follow-up from facilitators and “could have always reached out [to facilitators] if you had a question. They were they were always there for you…if you need to talk to me or whatever, or you can give me a text or email’ and then she’ll follow up when she’s got a moment”.

#### Subtheme 5c actions to address their needs

Participants discussed various ‘actions’ initiated by facilitators and other peer support recipients in efforts to comfort them and address their needs. Physical touch/connection needs were met virtually through verbal expressions (e.g., stating ‘virtual pat on the back’ or ‘virtual hug’) or emojis (e.g., thumbs up to indicate agreement), although PSR4 felt that these were not the same as physical expressions: “Somebody wants to hug, I just say virtual hug. But some people I have known for a long time from peer support meetings and sometimes I really feel love for them ‘cause they’re going through a lot of hard times.” In addition, participants shared and received advice and resources to alleviate their challenges.

The community program actually who helped me. Some of my family was coming to visit and I was really struggling with that…one of them in particular was able to say to me, this is an opportunity for you to help educate them on why you’re not able to do the things that you were able to do. Yeah, so we practiced in the program some of the things I could say. And the next week when I came back, I had had my sister up on that weekend and I was very thankful and I said it really went well and I was able to use some.

However, as noted by S8 (peer support recipient), there was a limit to the amount of help that could be provided, and it was necessary for others to remain within the scope of one’s ability/knowledge:

You certainly can’t start trying to solve their depression mental issues. You know that that’s almost a compassion reverse. You had to totally back off that let them talk. They wanna talk about it, OK, maybe I can say a few things that I knew some people, but you couldn’t. Let’s start if if you start trying to solve their problems. That’s not compassion. That’s almost torture. You, you’re gonna might lead them in the wrong direction…Opposite of compassion, you think you’re offering compassion by helping them but the problem is beyond the scope of what I can do.

Finally, facilitators, such as B1 (peer support recipient and provider) demonstrated compassion by modifying programs based on participants’ identified needs and preferences:

I remember sending emails to the coordinator in terms of saying to her I think this is a discussion point…in a few weeks and said hey, we heard this concern, come up and seem to be a lot of people interested in dialoguing about this. So let’s intentionally talk about this this week. So I think I think they were they were compassionate in that they were listening in and said, hey, there’s there’s more more food here to talk about, so let’s be let’s talk about this.

### Theme 6 compassion-related program outcomes

Peer support recipients identified compassion as a “required” (PSR6) component of virtual peer support stroke programs. The impact of compassion was both direct and indirect, as evidenced by alleviating the challenges faced by peer support recipients (subtheme 7a), enhancing their wellbeing (subtheme 7b), and enhancing their support (subtheme 7c).

#### Subtheme 6a alleviating challenges

Peer support recipients, such as PSR1, indicated that participating in a program lacking compassion would be an ineffective use of their time: “when there’s no compassion, you’re not wanting gonna want to participate, or not gonna want to go or not want to open up. You get feeling like they’re not really there there. Just posing as something like, you know that wasting, wasting your time is what I say it’s called.” Participants stated that compassion in the programs motivated them to continue attending, as they felt comfortable asking questions and openly sharing. Sharing their experiences allowed them to receive advice and resources from others that alleviated their challenges and met their needs. Additionally, S5, a peer support provider, explained that by being compassionate towards oneself and others, recipients could find hope in the stories and experiences of others, thereby reducing their perceived challenges:

The peer support person goes ‘I’ll never be able to do that.’ And I said ‘Yes, you will. You just have to sit and think on it for a minute.’ If you look for hope, you can find compassion. Because in doing so, you have to be compassionate to find hope. Otherwise, you write the person off.

However, some participants found that the policies and restrictions within the virtual programs presented challenges. For example, PSR9, a peer support recipient, believed that restricted topics reduced compassion as some information he wanted to share that was restricted could have helped others:

We weren’t allowed to talk about medicines and stuff like that…A chiropractor, ancient Chinese Medicine. They didn’t want to hear anything about that ‘cause it’s your own private thing…[but] maybe it may help somebody.

In addition, S1, a peer support recipient, indicated that privacy restrictions prevented her from addressing the needs of another peer support recipient, which reduced perceived compassion in the program. Some participants also noted that direct messaging with other peer support recipients was disabled on virtual platforms, preventing them from directly contacting other recipients.

One of the people participating yesterday is having difficulty and doesn’t have a lot of finances to get the things she needs. So a number of us said we have things we would like to give her that we no longer need but they can’t release information.

#### Subtheme 6b enhancing wellbeing

There was an indirect benefit of compassion in enhancing wellbeing. Compassionate programs motivated participants to continue participating in the virtual peer support stroke program, resulting in improved wellbeing by providing opportunities to connect with others. In addition, it validated and normalized their experiences and concerns. For example, S8, a peer support recipient, learned about an accessible skiing program from another peer support recipient, which was a valuable hobby for him before his stroke: “[the program] is offered in, near, well near Collingwood…I had no idea about it. And they help people with disabilities that got various equipment that can help you actually ski, which I was an avid avid skier.” S5, a peer support provider, highlighted the positive impact of compassion in her experience of providing support to others as she could help them visualize their future, providing hope and a roadmap for recovery: “I had a mentor who helped me as well with the roadmap and provided hope to me, so that’s something I want to do for others. That’s compassion right there”.

#### Subtheme 6c enhancing support

Participants emphasized the importance of compassion in virtual peer support stroke programs, indicating that they could not have benefited from programs that they felt were not compassionate. As PSR3, a peer support recipient, stated, “Well, if there is no compassion, then one is there.” According to participants, the program provided support, including developing new friendships with others across Canada. These friendships were valuable because participants’ family and friends could not always relate to their experiences of living with a stroke. In addition, an environment/space where compassion was promoted led to participants feeling comfortable seeking support and learning from others. As S1, a peer support recipient, explained, “I think that if it if it wasn’t compassionate, it wouldn’t have been a good experience at all. So, it turned, uh, it ended up being a like a a relevant experience for me and gave me an opportunity to ask questions I probably never would have asked before.” PSR3, (peer support recipient, echoed these sentiments, stating: “You can’t really be supportive without compassion”.

## Discussion

This study provides a valuable contribution to the literature, as it is, to the best of our knowledge, the first qualitative study to describe peer support providers’ and recipients’ (n = 24) experiences of compassionate support in virtual peer support stroke programs. Our study findings highlight the importance of compassionate support in virtual peer support stroke programs, as receiving such support is essential for peer support recipients to continue participating in these programs.

Researchers have cautioned against considering compassion to be an abstract concept as that can make it difficult to implement [55]. Thus, our study explicitly identifies facilitators and barriers to compassion within virtual peer support stroke programs (Table 4) using the empirical model of compassion [27]. These findings have implications for optimizing the design and delivery of compassionate virtual peer support stroke programs, including the training provided to peer support providers and program staff.

Our study contributes to the existing literature on compassion, which has primarily focused on compassion in health services [27, 56] or virtual care delivered by healthcare providers [22]. A novel aspect of our study is that we explored compassion from the perspectives of peer support providers and recipients who had a stroke and those that were both recipients and providers, allowing us to capture two orientations of compassion, including that felt towards others and that received from others [57]. Although the empirical model of compassion [27] was developed in the palliative care context, our analysis suggests that an adapted version of this model is also relevant for the context and population of our study. Guided by this model, we examined various dimensions of compassion from the perspectives of peer support providers and recipients, including the relational aspects that can be facilitated or hindered in a virtual environment. While the concept and themes of compassion generally align with existing understandings of compassion [27, 57, 58], we identified subtle nuances particularly relevant to virtual peer support stroke programs and adapted the model to fit with this context. For instance, we identified specific considerations for the stroke population in terms of compassionate support, such as the need to tailor facilitation techniques to support the engagement of individuals with aphasia or cognitive impairment. Additionally, we found that peer support recipients could establish connections, rapport, and relationships with peer support providers based on their shared stroke-related impairments, including functional challenges. In contrast, we found that when health providers without lived experience deliver services, building the necessary rapport to engage meaningfully with individuals may take longer. Finally, we found that compassion as a response to “address the *suffering* and needs of a person” [27] did not resonate with some participants who preferred the term ‘challenge’ rather than ‘suffering’ due to the negative connotations of suffering [59–61]. In sum, our study builds on the existing model of compassion [27] to understand its components within the studied context.

Barriers to compassion in a virtual program aligned with prior research findings. For instance, large group sizes could hinder compassion as it decreases recognition of participant needs and how prioritized they feel [22]. Other barriers, such as distrust due to security or privacy concerns, may compromise an individual’s ability to be open to receiving compassion from others [62]. The barriers to compassion identified in our study are important to address because there is considerable evidence that compassionate support impacts resilience to distress and one’s physical and mental health [22, 57].

Based on the study findings and existing literature, we have presented several provider/facilitator- and organizational-level recommendations to promote compassion in virtual peer support stroke programs.

### Implications for practice for peer support providers and facilitators of virtual peer support stroke programs

Peer support facilitates compassion by enabling a sense of connectedness and understanding about shared lived experiences [22]. In addition, actively listening is essential for facilitators in virtual programs as it fosters a compassionate presence, where facilitators are fully present with deep attention and non-judgmental listening, allowing the other person to feel comfortable engaging [56, 63]. Positive virtues and emotions of a person, including being calm, present, and kind, are recommended to demonstrate compassion towards others [58]. These virtues and emotions could be sensed by others [58] based on the other person’s non-verbal cues [64]. Picture-in-picture functionality of platforms can be helpful for facilitators to monitor their non-verbal cues, such as facial expressions [22]. Facilitators should be attentive to the differing communication needs of individuals with stroke and leverage tools (e.g., chat function) and tailoring strategies to enhance accessibility and meet their needs [22]. Enabling these approaches requires having a thorough understanding of how to use the technology and reducing technology-related barriers which can hinder compassion [63]. The promotion of optional anonymity (e.g., de-identifying program participants) [22] was not identified in our study. However, it could be a strategy used to explore to alleviate security concerns for some people, as security concerns are common among participants of virtual programs [63] and can prevent them from being open to receiving compassion from others [57]. Altogether, implementing these recommendations can help create a space for active and meaningful engagement for giving and receiving compassion.

### Recommendations for organizations that provide virtual peer support stroke programs

Organizations may need to focus training efforts towards helping peer support providers and facilitators foster compassionate listening skills [58] including how to have deep and active listening skills, feel empathy for others [33], and develop compassionate nonverbal communication skills [63], which can increase compassion towards others and connectedness [22, 58]. Virtual training [63] may also be helpful to those less familiar with the platform. While some training models already exist and may be adapted to fit various organizational contexts, there is an identified need for training approaches for nonverbal communication, particularly for those with communication difficulties [63, 65]. Finally, objective measures to measure the degree of compassion provided towards others and felt by others is also recommended [57].

### Limitations and strengths

Our study also has some limitations. First, this study was not representative of the patient population. For example, peer support providers’ perspectives were underrepresented, as most participants in this study were recipients. In addition, the perspectives of gender-diverse individuals (i.e., those who identified as other than men and women) were not represented in this study. Second, the study context should be considered. Participants were primarily recruited from March of Dimes in Canada, a national non-profit community-based organization that delivers stroke services. In addition, since the qualitative interviews were conducted virtually, participants were likely those with adequate internet connectivity, and as such, we were unable to capture technical challenges that may impact compassionate support in a virtual program. Lastly, we did not collect data on the duration of participants’ involvement in a virtual program, which may impact their experiences of compassionate support (e.g., a longer duration within a program could potentially foster heightened perceptions of compassion). Future research should consider implementing our recommendations to determine their utility and compare compassion within in-person, hybrid and virtual delivery of peer support stroke programs.

Despite these study limitations, we continued recruiting participants until we achieved data sufficiency to address the research questions. Semistructured interview questions were developed based on the empirical model of compassion [27], which allowed us to explore specific components of compassion while allowing the participants to direct the discussion. Methodological rigour was enhanced by involving multiple researcher team members in the data collection and analysis stages [66]. We also used a hybrid analysis approach that allowed us to tailor the model to fit the unique study context.

### Conclusions

This qualitative study comprehensively describes the perspectives of 24 peer support providers and recipients regarding compassionate support in virtual peer support stroke programs. Our study findings suggest that compassion resulted from the virtues of facilitators (i.e., genuineness, passion, and empathy), the virtual space, and communication within the virtual peer support stroke program (e.g., sense of awareness or intuition of compassion, aspects of engaged peer support provision), virtuous response (e.g., knowing the person and actions that made the peer support recipient feel like a priority). This study identifies several facilitators to compassion within virtual peer support stroke programs, including listening and understanding peer support recipients’ needs as they relate to stroke, attending to peer support recipients’ needs, and achieving compassion-related program outcomes. An absence of these factors hindered compassion in these programs. The practical recommendations for peer support providers, facilitators, and organizations that deliver virtual peer support stroke programs may help improve compassion within virtual peer support stroke programs. With the growing prevalence of virtual peer support stroke programs, our study contributes comprehensive and practical insights into an understudied research area, which can potentially improve the delivery of compassionate support within virtual peer support stroke programs.

## Supporting information

S1 Appendix Interview guides. (DOCX)
