# Supplementary material for: Peer support provider and recipients’ perspectives on compassion in virtual peer support stroke programs: “You can’t really be supportive without compassion”
Source: PLoS One. 2024 Oct 4;19(10):e0309148. doi: 10.1371/journal.pone.0309148 (PMC11451998; doi:10.1371/journal.pone.0309148)
Supplement: S1 Appendix — (DOCX) [file pone.0309148.s001.docx]

**Supplementary materials-Interview guides**

**Peer-support recipient**

- 1. Please tell me about the peer support program you were part of?
     1. *group or individual [if attended both, ask how they were different] What platform was it delivered on?*
  2. Why did you join the online peer support program?

1. What information did you share during the program?
2. What information did the peer-support provider share with you?
   - 1. How was this information helpful?
     2. What information would you have liked to hear more of?
   1. How comfortable were you with the peer-support provider in an online environment?
      1. How would it have been different in an in-person environment?
   2. What helped you feel more comfortable getting peer support in an online environment? How connected do you feel to the peer support provider? (e.g., on a scale of 1-10, 1 = not connected at all; 10 = very connected)
      1. Please explain this rating. How could this be changed to a 10?
3. How did the peer support provider show their empathy or concern for your wellbeing? (e.g. video, voice, anything to connect with you?
4. How comfortable were you sharing your concerns during the online peer support?
   - 1. What could have helped you feel more comfortable during the online peer support?
5. Could you please describe any examples of whether you felt unheard in the program?
   1. Please tell me how you felt
   2. What strategies could help you feel more supported/heard?
6. What does compassionate online program look like to you?
   1. Could you please describe an example of when you received compassionate peer support (e.g. feeling acknowledged and heard in the program)?
   2. Please share situations where you felt heard/listened to during the online peer support?
      1. What could have helped you feel more heard/listened to?
   3. Please share examples of situations where you felt valued during the online peer support?
      1. What could have helped you feel more valued?
   4. How did you build connections with others during the online peer support? (e.g. log on early)
      1. What could have helped you feel more connected to others?
7. **Compassion** can be defined as a state of concern for the unmet need of another, coupled with the desire to alleviate that.” ^10^
8. On a scale from 1-10, how compassionate was this program?
   1. Please share 3 things that made this program compassionate?
      1. Ask them to define the term or give examples – ask can you please tell me what that word means to you? Do you have any examples that demonstrate this
   2. Please share 3 ways that the online peer support could have been more compassionate? Please share 1 example of how people in the online program showed a concern for your needs?
      1. What did that look like? Probe about actions, words used, feelings
   3. Please share examples of any concerns or needs you had when received peer support?
      1. How did the peer-support provider alleviate your concerns?
9. What advice would you give a new peer support provider to help them be more compassionate?
10. What does a non-compassionate peer-support program look like?
11. What is the impact of compassion in peer support programs?

**Peer-support provider**

1. Please tell me about the peer support program you were part of?
   1. group or individual [if attended both, ask how they were different] What platform was it delivered on?
2. Why did you join the online peer support program?
3. What does compassionate online peer support look like to you?
4. Please tell me about your how it was to provide peer-support in the virtual peer-support stroke program.
   1. What was your relationship with the members like?
      1. How would it have been different in an in-person environment?
   2. How comfortable/connected did you feel with the members?
      1. How would it have been different in an inperson environment?
   3. How did you make members feel heard/listened to in the online peer support program?
      1. Was there anything that prevented members from feeling heard/listened to in the program?
   4. How did you make members feel valued in the online peer support program?
      1. Was there anything that prevented members from feeling valued in the program?
   5. How connected did you feel to the peer support recipients (e.g., on a scale of 1-10, 1 = not connected at all; 10 = very connected) Please explain. How could this be changed to a 10?
5. **Compassion** can be defined as “a state of concern for the suffering or unmet need of another, coupled with the desire to alleviate that suffering.” ^10^
   1. What does it mean to provide compassionate peer support?
6. On a scale from 1-10, how compassionate was this program?
   1. What are 3 things that made this program compassionate?
   2. Can you please provide an example of how you got to know the peer-support recipient’s needs/concerns?
      1. What did you do to address their needs/concerns?
   3. What are 3 things that prevented this program from being compassionate?
   4. How could this program be more compassionate?
7. Could you please describe any examples of providing compassionate peer support (e.g. feeling acknowledged and heard in the program)?
   1. What was said, what were they doing, what else was happening
   2. Please tell me how you felt
8. What does uncompassionate peer support look like?
   1. What was said, what were they doing, what else was happening
   2. Please tell me how you felt
   3. What strategies could help you feel more supported/heard?
9. What advice would you give a new peer support provider to help them be more compassionate?
10. What is the impact of compassion in peer support programs? (e.g. on mental health)
